# Development of a Highly Differentiated Human Primary Proximal Tubule MPS Model (aProximate MPS Flow)

**DOI:** 10.3390/bioengineering11010007

**Published:** 2023-12-21

**Authors:** Francesca Pisapia, Donovan O’Brien, Elena Tasinato, Kathryn L. Garner, Colin D. A. Brown

**Affiliations:** 1 Newcells Biotech Ltd., The Biosphere, Draymans Way, Newcastle Helix, Newcastle upon Tyne NE4 5BX, UK; donovanob@gmail.com (D.O.); elena.tasinato@newcellsbiotech.co.uk (E.T.); colin.brown@newcellsbiotech.co.uk (C.D.A.B.); 2 Institute of Genetic Medicine, Newcastle University, International Centre for Life, Central Parkway, Newcastle upon Tyne NE1 3BZ, UK

**Keywords:** human proximal tubule epithelial cells, primary cells, flow, micro-physiological system model, fluid shear stress, trans-epithelial electrical resistance

## Abstract

The kidney proximal tubule (PT) mediates renal drug elimination in vivo and is a major site of drug-induced toxicity. To reliably assess drug efficacy, it is crucial to construct a model in which PT functions are replicated. Current animal studies have proven poorly predictive of human outcome. To address this, we developed a physiologically relevant micro-physiological system (MPS) model of the human PT, the aProximate MPS Flow platform (Patent No: G001336.GB). In this model, primary human PT cells (hPTCs) are subjected to fluidic media flow and a shear stress of 0.01–0.2 Pa. We observe that these cells replicate the polarity of hPTCs and exhibit a higher expression of all the key transporters of *SLC22A6* (OAT1), *SLC22A8* (OAT3), *SLC22A2* (OCT2), *SLC47A1* (MATE1), *SLC22A12* (URAT1), *SLC2A9* (GLUT9), *ABCB1* (MDR1), *ABCC2* (MRP2), *LRP2* (megalin), *CUBN* (cubilin), compared with cells grown under static conditions. Immunofluorescence microscopy confirmed an increase in OAT1, OAT3, and cilia protein expression. Increased sensitivity to nephrotoxic protein cisplatin was observed; creatinine and FITC-albumin uptake was significantly increased under fluidic shear stress conditions. Taken together, these data suggest that growing human PT cells under media flow significantly improves the phenotype and function of hPTC monolayers and has benefits to the utility and near-physiology of the model.

## 1. Introduction

The proximal tubule (PT) is the region of the kidney nephron where more than two-thirds of the glomerular ultrafiltrate is selectively reabsorbed (e.g., small molecules, water, ions, drugs) [1]. Therefore, the PT is constantly exposed to high concentrations of drugs and metabolites that can exert their toxic effects on different targets in the PT cells through different mechanisms, thus leading to tubular cell toxicity [2]. For this reason, the PT is of particular interest for nephrotoxicity studies. Moreover, due to their proximity to the glomerulus, PT cells (human proximal tubule cells, hPTCs) are constantly exposed to the mechanical forces resulting from the flow of glomerular filtrate which induces fluid shear stress at the apical cell surface. This fluid dynamic stimulus regulates several functions of renal epithelial cells, including barrier function, transporter expression, cytoskeletal remodeling, protein uptake, and transport. Previous studies have shown that cellular responses to shear stress occur at several levels, evident through the altered expression of the genes involved in the cell matrix, cytoskeleton, and glycocalyx remodeling as well as metabolomic alterations [3]. Primary cells grown in vitro gradually lose features characteristic of the in vivo phenotype. Previous work has shown that after seven days in culture, renal proximal tubules can have less than 25% of their transporter expression relative to freshly isolated proximal tubules [4]. Factors which drive this phenotypic change can include, but are not limited to, an altered growth substrate (plastic dish), oxidative stress, an altered biochemical microenvironment, and a loss of paracrine signaling. It has been demonstrated that recapitulating the biomechanical cues to which they are exposed in vivo can improve viability, the formation of tight junctions, the gene expression of hPTC-specific markers, transporters, and endocytic receptors [5,6,7]. To address this, culture techniques that help to maintain cell-specific phenotypes are advantageous for the development of a more advanced model.

Although animal experiments remain the gold standard in drug development and the preclinical evaluation of novel compounds, recent studies have highlighted the inaccuracy and poor predictability of these models due to species-specific differences between these models and humans [8]. In recent years, there has therefore been an expansion in physiologically relevant micro-physiological systems (MPS) of PTs to recapitulate differentiation and function in vitro [9,10], with the aim of reducing the reliance or need for testing on animals while creating better models simultaneously. Moreover, the recent passing of the FDA Modernization Act 2.0 by the U.S. Congress, which removes the requirement for animal testing in drug development, opens the way for adopting alternative models that faithfully replicate human biology. One of the most promising technologies within the field is that of so-called “organ-on-chip” (OOC) platforms wherein cells from specific organs, which are either immortalized or primary- or stem cell-derived, are placed in 3D cell culture environments in which specific elements of the in vivo environment, such as biophysical stimuli and tissue architecture, are simulated to improve cellular phenotype and thus their relevance. In particular, polydimethylsiloxane (PDMS)-based microfluidic OOC systems have emerged with the goal of mimicking important physiological and mechanistic aspects of organs in vitro. PDMS has become the substrate of choice due to its ease of use, optical transparency, and inexpensive fabrication, but these systems are not without limitations. In particular, the substrate has a high absorptive affinity for hydrophobic small molecules and combined with a lack of scalability for high-throughput studies, these shortcomings render these systems inapplicable to drug discovery, in vitro pharmacokinetic (PK) modeling, cellular mechanistic studies, and personalized medicine. Similarly, these systems often do not allow for the translatability of established cell culture techniques and can require expensive equipment to operate.

As a result, our aim was twofold. Firstly, to fabricate a biomimetic system which was able to effectively recapitulate key biophysical stimuli, but which also was fabricated from non-absorptive materials and could be scaled up to accommodate high-throughput studies. Secondly, to investigate whether the introduction of shear stress improves our well established static aProximate human PT cell model to deliver an improved and more realistic platform for drug efficacy, drug development, and drug toxicology studies.

Here we report the fabrication of our aProximate MPS Flow human PT platform in which primary hPTCs are subjected to fluid shear stress (FSS) using a rocker device that allows a calculated level of shear stress to be applied to the cells under culture. Indeed, by performing computational fluid dynamic (CFD) analyses, we were able to determine the optimal shape and dimensions of the flow channel to achieve maximal, uniform shear stress exposure. Using a bio-compatible photopolymer resin for the printing of the device, we generated a prototype system for use with existing 24-well insert systems, which is suitable for the simulation of key micro-physiological stimuli and modeling drug uptake and cellular response without losing scalability or predictability due to physical material limitations.

## 2. Materials and Methods

### 2.1. aProximate MPS Flow Design

**Figure 1:**
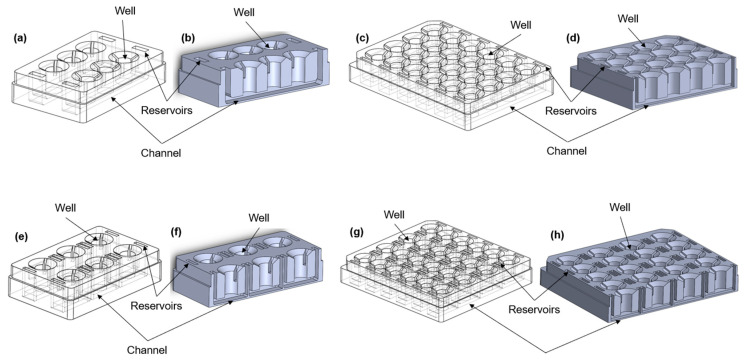

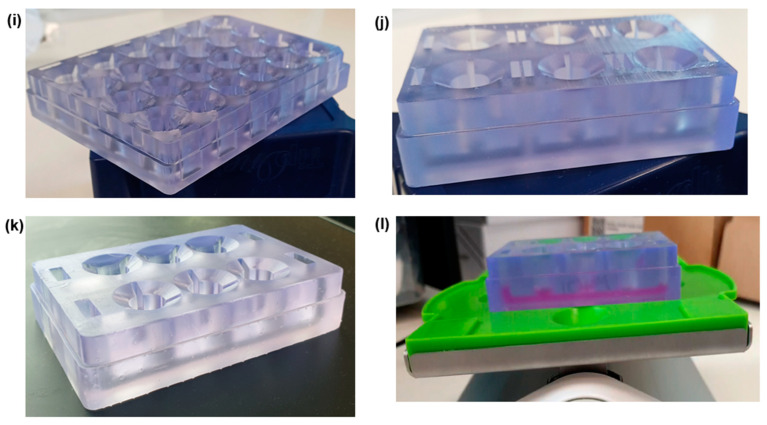
Different configurations of the aProximate MPS Flow human PT platform. Isometric views (left) and cross-section views (right) of (**a**,**b**) the 6-well format, where each row consists of three wells exposed to the same connected fluid flow; (**c**,**d**) the 24-well format, where each row consists of four wells and is exposed to fluid flow; (**e**,**f**) the 6-single well format, where each well is individually exposed to fluid flow; (**g**,**h**) the 24-single well format, where each well is individually exposed to fluid flow; (**i**) picture of the 24-well format; (**j**) picture of the 6-signle well format; (**k**) picture of the 6-well format; (**l**) experimental setup showing the MPS system with media on a rocker platform.

The microfluidic device was designed using SolidWorks software, version SP0, 2022 (Dassault Systems, Vélizy-Villacoublay, France), in different configurations (Figure 1a–h). All of the formats consist of a number of wells into which a 24-well size semi-permeable cell culture insert can be placed to create upper and lower chambers. In some of the configurations (Figure 1a–d), the lower chambers are connected to each other through a channel to allow the movement of the medium between the chambers. Cells are grown on the underside of the insert and are exposed to the resultant shear stress set up in the lower chamber. The device was printed using a stereolithography (SLA) printer (Formlabs, Somerville, MA, USA) from a biocompatible [11] liquid photopolymer resin (Formlabs BioMed Clear Resin) and then washed in 90% isopropyl alcohol (IPA) for 25 min (Formlabs Form Wash) to remove excess resin. Once dry, the device was cured in UV light (Formlabs Form Cure) to enhance the mechanical properties. The device was sterilized according to the photopolymer resin protocol established in previous studies [12,13,14]. The device was washed twice in Dulbecco’s phosphate buffered saline (DPBS, 14190144, Gibco, New York, NY, USA) for 5 min before completely immersing the channels in 70% ethanol for 10 min. Finally, the device was washed using sterile distilled water and UV-sterilized for 30 min.

### 2.2. Computational Fluid Dynamic Simulations

**Figure 2:**
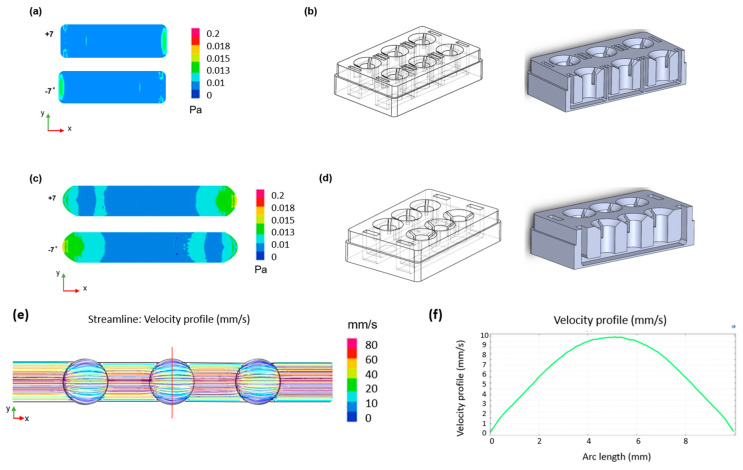
CFD simulations of the designed device. Two-dimensional shear stress distribution within (**a**) the single well format channel with (**b**) relative geometry and (**c**) the connected well format channel with (**d**) relative geometry during the oscillatory rocking movement at ±7°. (**e**) Two-dimensional velocity distribution across the channel and (**f**) velocity profile across the width of the chamber (red line in (**e**)) for a shear stress of 0.02 Pa.

COMSOL Multiphysics 5.5 (COMSOL Inc., version 5.5, 2020, Stockholm, Sweden) was used to simulate the fluid flow within the device channels. The laminar low interface in the fluid flow module was coupled with the moving mesh interface in the mathematics module. The study models the fluid motion with the incompressible Navier–Stokes equations. The fluid is initially at rest in the channel. The motion is driven by the gravity vector (Equations (1) and (2)) swinging back and forth, pointing up to seven degrees away from the downward y direction.

(1) $$\;F_{x}=\rho \mathrm{gsin}\left(\theta_{\max}\sin\left(2\pi \Omega t\right)\right)$$

(2) $$F_{y}=-\rho \mathrm{gcos}\left(\theta_{\max}\sin\left(2\pi \Omega t\right)\right)$$

where g = 9.81 m/s^2^, θ_max_ = 7°, and Ω = 0.1 CPM. As the surface of the fluid is free to move, the arbitrary Lagrangian-Eulerian (ALE) method was set up using the moving mesh (ALE) interface. Slip boundary conditions (Equation (3)) were applied to the solid walls as follows:

(3) u·n=0

where n = (n_x_, n_y_)^T^ is the boundary normal. A free boundary condition was used for the fluid (top boundary). To follow the motion of the fluid with the moving mesh, the mesh motion was coupled to the fluid motion in the normal direction. The boundary condition for the mesh equations on the free surface is as follows:

(4) $${\left(x_{t},y_{t}\right)}^{T}\cdot n=u\cdot n$$

where n is the boundary normal and (n_x_, n_y_)^T^ the velocity of the mesh. At the bottom of the channel the mesh displacement was set to zero. The results are shown in Figure 2.

### 2.3. Cell Culture and Seeding

Primary hPTCs (aProximate) were isolated from fresh human kidneys as described previously [4] and maintained in a renal epithelial growth medium (REGM; Lonza, Basel, Switzerland) supplemented with renal epithelial growth factor supplements (Lonza, CC-4127). Once isolated, the cells were seeded at a concentration of 1 × 10^6^ cells/mL on the underside of a 24-well size cell culture insert (24-well ThinCert^®^ 0.4 μm pore diameter transparent, 662641, Greiner Bio-One Ltd., Gloucestershire, UK) and left overnight at 37 °C in a humidified 5% CO_2_ incubator to allow for attachment. The next day, the cell culture inserts were flipped into their standard configuration in the 24-well cell culture plate and growth medium was added to both the upper and lower chambers. The cells were grown in static culture for three days (until 80% confluent) before being placed into the sterilized microfluidic device. Growth media were then pipetted into the channel with 3 mL for the 6-well and 24-well formats and 800 µL for the 6-single well and 24-single well formats. The device was subsequently placed onto a rocker platform which, by tilting it at a fixed frequency (0.1 cycles/min), allowed periodic bi-directional gravity-induced movement of media from one chamber to the other, thus exposing the monolayer of cells to a basolateral fluid shear stress (~0.02 Pa), mimicking that experienced by hPTCs in vivo [15,16].

### 2.4. Barrier Formation

Transepithelial electrical resistance (TEER) was measured using an EVOM^2^ ohmmeter with STX-2 probe (World Precision Instruments (WPI), Sarasota, FL, USA). Permeability was assessed using Lucifer Yellow CH dilithium salt, 10 mM in dH_2_O (Sigma Aldrich, Burlington, MA, USA). The apparent permeability (*P_app_*) was measured using Equation (5) [17] where *V_R_* is the volume of the receiver compartment, *dC_R_*/*dt* is the change in the drug concentration of the receiver compartment over time, *A* is the surface area of the membrane (0.3 cm^2^), and *C_D_*_0_ is the concentration of the drug in the donor compartment at time zero.

(5) Papp (cmmin)=VR∗dCRdt∗A∗CD0

### 2.5. RNA Isolation and Reverse Transcription

Primary hPTCs were lysed with 350 μL RLT lysis buffer (QIAGEN GmbH, Hilden, Germany) and stored at −80 °C overnight. RNA was isolated using the RNeasy PLUS Mini Kit (QIAGEN GmbH, Hilden, Germany) following instructions provided by the manufacturer and quantified using a spectrophotometer (NanoDrop Technologies, Thermo-Fisher Scientific, Wilmington, DE, USA). The mRNA (400 ng) was reverse transcribed to complementary DNA (cDNA) according to the instructions provided by the manufacturer using MMLV reverse transcriptase (Promega, Madison, WI, USA), RNasin (Promega, Madison, WI, USA), dNTPs (Promega, Madison, WI, USA), Random Primers (Promega, Madison, WI, USA), and a SimpliAmp thermocycler (Applied Biosystems Ltd., Waltham, MA, USA).

### 2.6. Quantitative Real-Time PCR

Expression levels of mRNA were determined using QuantStudio 6 Flex (Applied Biosystems, Waltham, MA, USA). Primers were designed using Primer-BLAST (NCBI, Bethesda, MD, USA). A GoTaq qPCR (Promega, Madison, WI, USA) kit was used to run qRT-PCR assays. Data were analyzed using QuantStudio Software version 1.7.2 (Applied Biosystems, Waltham, MA, USA). Gene expression was calculated as the ∆CT relative to the housekeeping gene GAPDH. The primer sequences are shown in Table 1.

### 2.7. Immunofluorescence Microscopy

hPTCs were washed three times with DPBS and fixed with paraformaldehyde (2%; Thermo-Fisher Scientific, Kandel, Germany) with 200 μL in the upper chamber and 600 μL in the lower chamber for 15 min at room temperature. The cells were washed again three times with DPBS and then permeabilized using Tween-20 (0.05% *v*/*v*; Sigma-Aldrich, St. Louis, MO, USA) in DPBS for 10 min. Next, the permeabilized inserts were incubated in blocking solution (bovine serum albumin) (1% *w*/*v*; Sigma-Aldrich, St. Louis, MO, USA), bovine serum (10% *v*/*v*; Sigma-Aldrich), and 0.05% Tween-20 in DPBS in a humidified chamber for 1 h. Primary antibody solutions were made by diluting antibodies in blocking solution and, following the removal of the blocking solution, were incubated with the cells overnight at 4 °C. The following primary antibodies were used: rabbit (Rb) anti-ZO-1 (Invitrogen, Cat # 61-7300), mouse (Ms) anti-acetylated tubulin (Merck, Rahway, NJ, USA, Cat # T7451), Rb pericentrin (Invitrogen, Waltham, MA, USA, Cat # 17108963) Rb SLC22A6 (Biorbyt Ltd., Cambridge, UK, Cat # orb136646), Rb SLC22A8 (Biorbyt Ltd., Cat # orb136646), rat (Rt) anti-sodium potassium ATPase antibody [EP1845Y] (Abcam, Cambridge, UK, Cat # ab283341). The next day, after washing the cells three times with DPBS, secondary antibodies were added and left for 1 h. The following secondary antibodies were used: goat anti-Rb Alexa Fluor 647 (Invitrogen, Cat # A21245), goat anti-Ms Alexa Fluor 488 (Invitrogen, Cat # A11001), goat anti-Rt Alexa Fluor 546 (Invitrogen, Cat # A11081). After another 3 washes, cells were incubated with 10 μg/mL Hoechst 33342 stain (Thermo-Scientific, Kandel, Germany). Finally, the membranes were cut out of the inserts and placed on microscope slides using SlowFade Gold (Invitrogen, Waltham, MA, USA) mounting media. Images were taken using a 20× magnification ZEISS AxioImager with Apotome (Zeiss, Jena, Germany) and intensity quantification was performed using ImageJ software, 1.54g.

### 2.8. Nephrotoxicity Assay

Cisplatin (cis-diamineplatinum (II) dichloride) (Sigma-Aldrich, Burlington, MA, USA) was made up in N,N-Dimethylformamide (Sigma-Aldrich, Burlington, MA, USA) to a stock concentration of 20 mM before being diluted in cell culture media to the appropriate concentrations. ATP was measured using the CellTiter-Glo^®^ Luminescent Cell Viability Assay (Promega, Madison, WI, USA). Meso Scale Discovery (MSD, Rockville, MD, USA) technology was used to quantify the biomarker release using multiplex immunoassays based on electrochemiluminescence.

### 2.9. Compound Uptake Assays

To assess compound uptake, several compounds were tested for specific receptors. FITC-Albumin (Sigma-Aldrich, St. Louis, MO, USA) was prepared at a stock of 500 mg/mL and diluted in Krebs buffer as appropriate. Uptake assay was run for 90 min at 37 °C, before cells were lysed using Cell Lysis Buffer (Invitrogen, Waltham, MA, USA) and fluorescence measured using ClarioStar Plus (BMG Labtech GmbH, Ortenberg, Germany) to determine compound uptake. For radiolabeled compound studies, ^14^C-Creatinine and ^3^H-Mannitol were prepared to a stock of 10 μM and dosed in 200 μL (upper chamber) or 800 μL (lower chamber) of Krebs buffer on the appropriate side to determine uptake of the compound. Uptake assay was run for 90 min at 37 °C before reaction was stopped at 90 min with ice-cold Krebs and membranes cut out of inserts and placed in scintillation vials immersed in Ultima GOLD (Perkin Elmer, Waltham, MA, USA) scintillation fluid and DPM measured using a Tri-Carb 2910TR liquid scintillation counter (Perkin Elmer, Waltham, MA, USA).

### 2.10. Statistical Analysis

Graphical and statistical analysis was performed using GraphPad Prism version 10 (San Diego, CA, USA).

## 3. Results and Discussion

### 3.1. In Vitro Proximal Tubule Barrier Formation under Static and Flow Conditions

**Figure 3:**
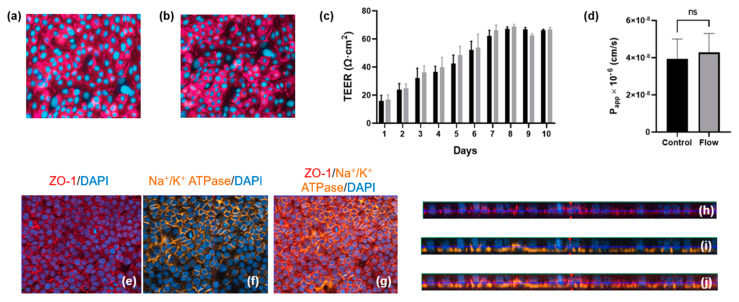
Barrier formation and integrity is comparable for aProximate cells grown under static and FSS conditions. (**a**,**b**) Tigh junction formation under static and flow condition, respectively; (**c**) TEER was monitored daily for 10 days for cells grown under static (black) and FSS (grey) conditions (data expressed as mean ± SD; representative experiment shown (*n* = 6)); (**d**) Apparent permeability (*P_app_*) was assessed using Lucifer Yellow up to 180 min on day 10 (data expressed as mean ± SEM; *n* = 6). Polarization and relative orthogonal projections confirm the apical localization of (**e**,**h**) tight junction formation (red) and (**f**,**i**) the basolateral localization of Na^+^/K^+^ adenosine triphosphatase (orange) for cells under FSS conditions; (**g**) Merged image of (**e**,**f**) and (**j**) merged image of (**h**,**i**).

Primary hPTCs were isolated from human kidneys and seeded onto the underside of 24-well cell culture inserts. The cells were allowed to adhere overnight before the inserts were turned the “right-way up” (standard configuration) and grown for three days until 80% confluent. At this point, the inserts were transferred to the aProximate MPS Flow system device and either cultured under static or fluid shear stress (FSS) conditions. Transepithelial electrical resistance (TEER) was assessed daily for the subsequent 10 days to monitor barrier formation and integrity. No difference was observed between cells grown under static (Figure 3a) or FSS conditions (Figure 3b); the cells maintained similar TEER values up to day 10 (Figure 3c). Barrier integrity was additionally measured on day 10 using a lucifer yellow permeability assay in which lucifer yellow reagent was added into the inserts and sampled from the lower chamber compartment after an incubation time of 180 min. Calculation of *P_app_* revealed no significant difference in barrier integrity between cells grown under static or FSS conditions (Figure 3d). The observed TEER of around 60 Ώ·cm^2^ and *P_app_* for Lucifer Yellow of around 4 × 10^−6^ cm/s were very close to the TEER values reported for in vivo proximal tubules of between 15 and 45 Ώ.cm^2^ [18] as well as the TEER values and *P_app_* for Lucifer Yellow of 12 × 10^−6^ cm/s reported for opossum kidney (OK) cells which are widely used as a model of proximal tubule barrier function [19]. There is little information available in terms of TEER and *P_app_* for Lucifer Yellow in other in vitro proximal tubule cells models grown either on filter supports or in MPS devices. Most recently, it was reported that conditionally immortalized (ciPTEC) cells grown on filter supports had a TEER of between 120 and 140 Ώ.cm^2^ [20]. Practically, a *P_app_* of around 1 × 10^−6^–1 × 10^−7^ cm^2^/s is ideal to differentiate between transcellular and paracellular transport of molecules. A higher *P_app_* for Lucifer Yellow would make it difficult to distinguish transcellular flux of a molecule from a large background paracellular flux.

Polarization and relative orthogonal projections of PTCs under FSS conditions were evaluated using the apical localization of tight junction proteins (Figure 3e,h) and the localization of Na^+^/K^+^ adenosine triphosphatase (ATPase) to the basal border and between neighboring cells (Figure 3f,i). Figure 3g,j shows the merged images of Figure 3e,f and Figure 3h,i, respectively.

### 3.2. Transporter Expression Is Upregulated by Fluid Shear Stress

**Figure 4:**
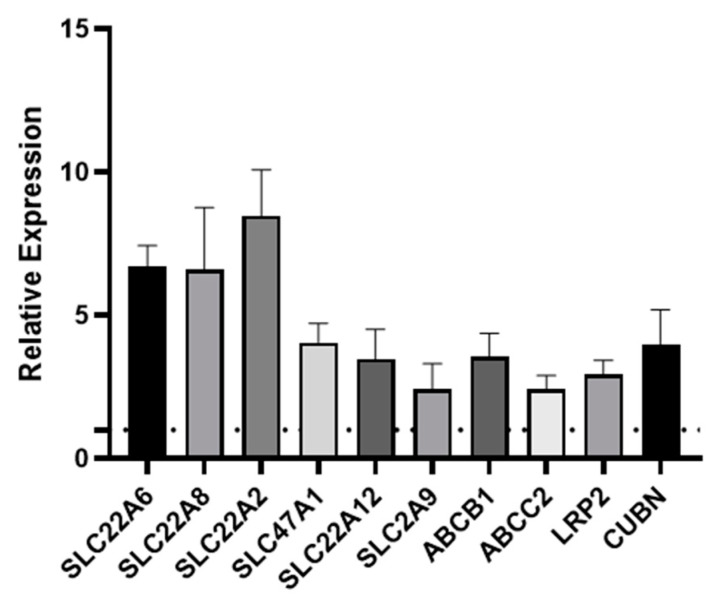
Fluid shear stress upregulates PT transporter expression. qRT-PCR was used to assess the expression of transporters following seven days’ culture under static or FSS conditions. *SLC22A6* (OAT1), *SLC22A8* (OAT3), *SLC22A2* (OCT2), *SLC47A1* (MATE1), *SLC22A12* (URAT1), *SLC2A9* (GLUT9), *ABCB1* (MDR1), *ABCC2* (MRP2), *LRP2* (megalin), and *CUBN* (cubilin). The data are expressed relative to the static control (dotted line) (mean ± SEM; *n* = 6).

**Figure 5:**
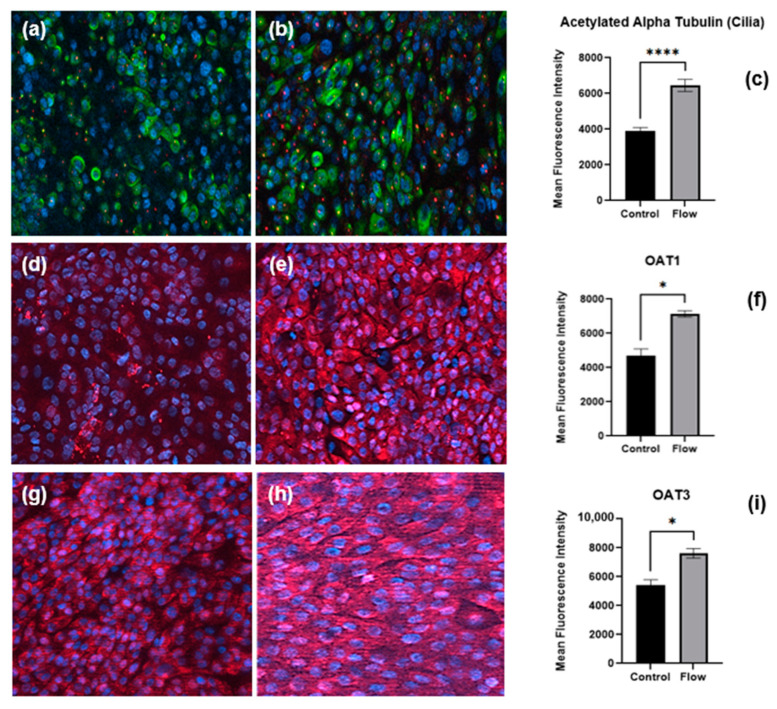
Immunofluorescence staining of proteins, cilia, and transporters of PTCs in static and flow conditions after seven days of culture. Staining of pericentrin (red) and acetylated α-tubulin (green) to visualize primary cilia in (**a**) static versus (**b**) flow conditions. Organic anion transporters expression (red) under static (**d**,**g**) and FSS (**e**,**h**) conditions, respectively. Quantitative analysis of mean fluorescence intensity of cilia (**c**), OAT1 (**f**), and OAT3 (**i**) (flow and control conditions were compared using Student’s *t*-test, **** *p* < 0.0001, * *p* < 0.05).

One of the key functions of the kidney is the selective reabsorption of more than two-thirds of the glomerular ultrafiltrate and the elimination of drugs, exogenous, and endogenous compounds from the organism. This elimination process occurs through transmembrane proteins which are responsible for both the secretion and absorption of the aforementioned compounds; therefore, it is important that an in vitro model of the PT expresses all of the key transporters. Transporters are expressed on either the basolateral or apical (luminal) membrane of primary hPTCs. The apical transporters are responsible for the efflux of substances into the tubular lumen, while the basolateral transporters ensure the absorption of substances from the bloodstream [21]. Over the years, we have reported the remarkable retention of differentiation in aProximate human PT cells grown on filter supports under static conditions. We have successfully shown the roles of OATs, MRPs, OATs, MDR1, megalin, and cubilin in the transport of a wide range of xenobiotics [22,23,24,25]. To investigate whether shear stress improved expression of these and other key transport proteins, we assessed the expression of ten key transporters in primary hPTCs after seven days culture under static or FSS conditions in the aProximate MPS Flow system. Seven days in culture was chosen as this was the timepoint we routinely used in our studies. We noted that all ten transporters were significantly upregulated under flow as follows: *SLC22A6* (OAT1), *SLC22A8* (OAT3), *SLC22A2* (OCT2), *SLC47A1* (MATE1), *SLC22A12* (URAT1), *SLC2A9* (GLUT9), *ABCB1* (MDR1), *ABCC2* (MRP2), *LRP2* (megalin), and *CUBN* (cubilin) at the mRNA level (Figure 4) or for a subset tested at the protein level (Figure 5).

We assessed the expression of ten key transporters in hPTCs after seven days culture under static or FSS conditions in the aProximate MPS Flow system. We noted that all ten transporters were upregulated under flow: *SLC22A6* (OAT1), *SLC22A8* (OAT3), *SLC22A2* (OCT2), *SLC47A1* (MATE1), *SLC22A12* (URAT1), *SLC2A9* (GLUT9), *ABCB1* (MDR1), *ABCC2* (MRP2), *LRP2* (megalin), and *CUBN* (cubilin) (Figure 4).

### 3.3. Cilia and Transporter Protein Expression Is Increased under Flow

Cells of the PT express cilia, one per cell, rich in acetylated α-tubulin and pericentrin proteins that project into the PT lumen containing the glomerular ultrafiltrate to sense fluid flow (apical surface) [15]. Primary hPTCs cultured under static or FSS conditions for seven days were fixed as well as immunofluorescence stained with antibodies against cilia proteins (Figure 5a,b); quantification revealed the upregulation of acetylated α-tubulin under FSS conditions (Figure 5c).

In order to confirm our observation that transporter gene expression is increased under flow, cells cultured under static or FSS conditions for seven days were fixed and underwent immunofluorescence staining for OAT1 (Figure 5d,e) and OAT3 (Figure 5g,h) renal organic anion transporters. Image analysis revealed the upregulation of both transporters under flow (Figure 5 f,i). The key observation of the expression of significant levels of OAT1 and OAT3 in both static and flow models is important. aProximate cells are perhaps unique in that they express OATs under static conditions and that the expression level can be enhanced by flow. It is widely reported that OATs are very labile in culture and are not expressed in in vitro immortalized HK2 PT or REPTEC/TERT1 cell models [26]. Indeed, to recapitulate OAT expression in ciPTEC cells requires the transfection of the cells with either OAT1 or OAT3 [27].

### 3.4. Primary hPTCs under Flow Are More Sensitive to Drug-Induced Nephrotoxicity

**Figure 6:**
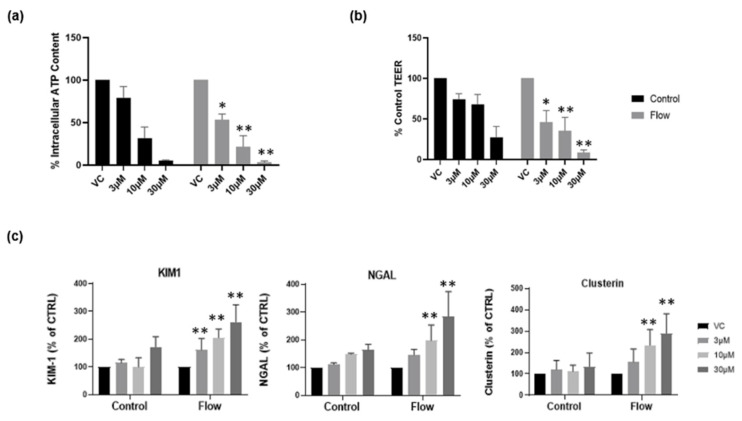
Cells grown under flow are more sensitive to nephrotoxic insult. Following seven days’ culture under static or FSS conditions, cells were exposed to cisplatin for 72 h. (**a**) ATP release and (**b**) TEER at day 10 in both static (black) and flow (grey) conditions; (**c**) renal stress biomarker release at day 10 (mean ± S; *n* = 4; flow and control conditions were compared using two-way ANOVA followed by Šídák’s multiple comparisons test * *p* < 0.05, ** *p* < 0.005). VC, vehicle control.

Recently, we reported that aProximate human PT cell monolayers grown under static conditions were highly predictive of in vivo drug-induced proximal tubule injury. We tested a panel of 36 xenobiotics with known in vivo renal potential to cause kidney injury using the biomarkers of KIM-1, Clusterin and NGAL together with TEER using ATP levels and LDH release as endpoints of damage. The outcome was that the aProximate model had a sensitivity of 60, a specificity of 94, and a positive predictive value of suggesting that the static aProximate model is a highly predictive model of kidney injury [4]. In this study, we postulated that an increase in transporter expression (particularly uptake transporters) might lead to an increase in xenobiotic exposure within the cell and an increase in toxicity at any one concentration compared with the static aProximate PT cell platform. To test this, cisplatin was administered to primary hPTCs under both static and flow conditions and alongside the impact on the end points of toxicity. KIM1, Clusterin, and NGAL together with TEER, ATP content, and LDH release were measured. The compound is a well-known chemotherapy drug; however, side effects include proximal tubule nephrotoxicity [28]. Cisplatin uptake is mediated via the basolateral transporter OCT2 as well as OAT1 and OAT3 [29,30,31]. Three different cisplatin concentrations (3 µM, 10 µM, 30 µM) were added to the cells cultured under both static and FSS conditions. For the cells cultured under static conditions, the nephrotoxic compound was added to the apical and basolateral compartment of the inserts, whilst for the cells under flow condition the compound was added to the apical compartment of the inserts and into the channel through one of the reservoirs. At 72 h following treatment, the biomarker release, the barrier function, and the cell viability were measured using an MSD multiplex assay, TEER measurement, and ATP assay, respectively. Figure 6a,b illustrates a drop in ATP release and TEER as the compound concentration increases. Moreover, cells under flow exhibit a higher sensitivity to the nephrotoxic compound compared with cells grown under static conditions (Figure 6c). This is an important observation as it implies that under flow conditions the assay will have the sensitivity to identify drugs that result in only small or non-significant perturbations in biomarker production that are not evident under static conditions.

### 3.5. Creatinine and Albumin Uptake Is Increased under Flow

**Figure 7:**
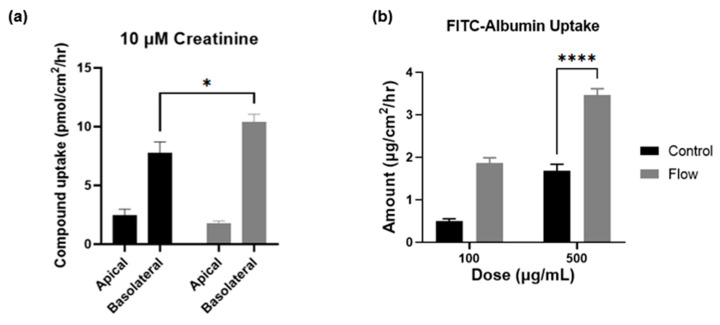
Creatinine and albumin uptake is increased under flow. Compound uptake of (**a**) 14-C labelled Creatinine and (**b**) FITC-Albumin at 90 min comparing static (black) and FSS-exposed cells (grey) (mean ± SD; *n* = 3; the two conditions were compared using a two-way ANOVA followed by Šídák’s multiple comparisons test * *p* < 0.05, **** *p* < 0.0001).

To measure the functional increase in transporter expression in cells exposed to FSS within the aProximate MPS Flow model and to further validate the gene expression findings, two compounds were selected for this study with discrete mechanisms of uptake. Creatinine is selectively taken into the proximal tubule through OCT2 on the basolateral membrane and is secreted using multidrug and toxin extrusion transporters 1 (MATE1; *SLC47A1*) and MATE2-k (*SLC47A2*) on the apical membrane [32]. Using a 14C-labelled creatinine tracing study, results illustrated significantly improved uptake of the compound by cells which had been exposed to FSS (Figure 7a). These results again imply that the aProximate MPS Flow model more closely mirrors the physiology of the in vivo tubule than the static model.

Albumin is taken into the proximal tubule via the endocytic receptors megalin and cubilin that play crucial roles in maintaining the integrity of the apical endocytic pathway for the efficient uptake of filtered proteins [33]. Indeed, a significant increase in the uptake of FITC conjugated albumin was observed in cells which had been exposed to FSS compared with static control at higher concentrations, thus reflecting the increases in gene expression levels of these receptors (Figure 7b). The importance of the functional expression of the megalin and cubilin complex is that receptor-mediated endocytosis plays a key role in the proximal tubule uptake of a wide range of large molecules such as albumin, antibiotics, antisense oligonucleotides, siRNA, and antibody and antibody fragments. Recent evidence suggests that the megalin-cubilin mediated endocytosis of these molecules is associated with proximal tubule injury [34]. Both the aProximate static and aProximate MPS Flow models have proved extremely useful in identifying these interactions.

## 4. Conclusions

In recent years, organ-on-chip technologies have provided novel and insightful methods for improving the quality and relevance of in vitro research. Specifically, within the field of toxicology, this has provided a marked improvement over the use of animal models for the analyses of responses within specific tissues wherein species-specific differences may be apparent. The use of PDMS to fabricate microfluidic chip systems represents two main obstacles for the field of toxicology. The first is that of throughput as, generally, PDMS chips are generated by casting molten PDMS onto the templates of chips and is not only a labor-intensive process but also a time-consuming one. Secondly, the use of PDMS in toxicology is highly controversial due to the absorptive properties it has of small molecule drugs.

Here, we describe a novel system that attempts to address both these concerns through taking advantage of a biocompatible, non-absorptive photopolymer to fabricate a multi-well system compatible with toxicological studies while still retaining uniform shear stress exposure across the membrane. We also attempted to circumvent the need for expensive control units or pumped systems for the generation of shear stress using a rocker system programmed to rock at a specific angle to generate an oscillatory fluid flow across the membrane.

A key concept underlying our design of the MPS plate was to develop a high throughput and a relatively low-cost platform based upon 24 and 96 transwell filter supports. There were several reasons to base our model on insert supports rather than on a pumped channel OOC design. These include the fact that inserts are a well-established format, designed for high throughput in either 24- or 96-well formats for which many automated assays are designed. They have a large surface area for cell growth (0.33 cm^2^), which makes them amenable to both radiolabeled and non-radiolabeled flux assays and to the quantification of biomarker releases for drug safety studies. In addition, the cell lysate is easily collected in quantities for a range of assays including miRNA and exosomes studies. In addition, optically transparent cell inserts are ideal for IHC and high-content screening.

Moreover, we describe both improved phenotypes and functions in primary hPTCs using this system. From an in vitro toxicological perspective, the upregulation of gene transcripts associated with key transporter proteins, such as those that comprise the OAT and OCT families as well as endocytic transporters like megalin and cubilin, ensures that the improved phenotype observed after the shear stress stimulation in our system is more representative in vivo; this was confirmed in our functional studies of transporter-specific drug targets and uptake assays, with cells having undergone exposure to shear stress showing marked improvements in both categories. To confirm protein-specific improvements, we utilized immunofluorescent imaging and cells which had been cultured within our system once again showed an improved expression of key proteins, further validating data generated from transcriptional experiments. Thus, by inducing fluid shear stress in the cell culture of our primary cell model using a novel fluidic device, the aProximate MPS Flow, we have illustrated an improved proximal tubule model on a transcriptional, functional, and phenotypical level.

Taken together, this dataset suggests that growing primary human PT cells on insert filter supports with media flow across the membrane significantly improves the phenotype and function of PT cell monolayers and has significant benefit to the utility and near-physiology of the model for toxicological and drug development studies.

## 5. Patents

The patent application is in progress (Patent No: G001336.GB).

## Figures and Tables

**Table 1:** Primer sequences used for the qRT-PCR for gene expression of key transporters.

Protein	Gene	Forward Primer Sequence (5′-3′)	Reverse Primer Sequence (5′-3′)
Organic Anion Transporter 1 (OAT1)	*SLC22A6*	ACCAGTCCATTGTCCGAACC	TGTCTGCCGGATCATTGTGG
Organic Anion Transporter 3 (OAT3)	*SLC22A8*	CACCGCAAGTGACCTGTTCC	CAGGAAGAGGGCAGCACTG
Organic Cation Transporter 2 (OCT2)	*SLC22A2*	ACCTGGTGATCTACAATGGCT	TGAGGAACAGATGTGGACGC
Multidrug And Toxin Extrusion Protein 1 (MATE 1)	*SLC47A1*	ATCGGGATCGCGCTGATGTT	TGTACCTGAGCCTGCTGACAGTCTGCCCACTCTGCACCTTC
Urate transporter 1 (URAT1)	*SLC22A12*	GTGTACTGCCTGTTCCGCT	CGTCCACTCCATCAGGAGA
Solute carrier family 2 member 9 (GLUT9)	*SLC2A9*	TCACAGATGACACCAGCCAC	ACAGGTTGTAGCCGTAGAGG
P-Glycoprotein 1 (MDR1)	*ABCB1*	TTCACTTCAGTTACCCATCTCG	GTCTGCCCACTCTGCACCTTC
Multidrug Resistance Associated Protein 2 (MRP2)	*ABCC2*	CACCATCATGGACAGTGACAAGG	CCGCACTCTATAATCTTCCCG
Lipoprotein receptor-related protein 2 (Megalin)	*LRP-2*	ATTGATGGCACAGGAAGAGA	GCTAGCCTCATGACACTGAT
Cubilin	*CUBN*	TGAAGGTGTGGGCAGGAAC	GAGACTGGAAGACGGCAGTG
Glyceraldehyde-3-Phosphate Dehydrogenase	*GAPDH*	TGACAACTTTGGTATCGTGAAGG	AGGCAGGGATGATGTTCTGGAG

## Data Availability

The data presented in this study are available on request from the corresponding authors.

## Abbreviations

CFD	computational fluid dynamic
DPBS	Dulbecco’s phosphate buffered saline
FSS	fluid shear stress
hPTCs	human proximal tubule cells
IPA	isopropyl alcohol
MPS	micro-physiological system
OAT1	organic anion transporter 1
OAT3	organic anion transporter 3
OCT2	organic cation transporter 2
OOC	organ-on-chip
*P_app_*	apparent permeability
PDMS	poly-dimethyl siloxane
PK	pharmacokinetic
PT	proximal tubule
qRT-PCR	quantitative real-time polymerase chain reaction
REGM	renal epithelial growth medium
SD	standard deviation
SEM	standard error of the mean
SLA	stereolithography
TEER	transepithelial electrical resistance
VC	vehicle control
ZO-1	zonula occludens 1
